# Postoperative Anticoagulation After Mitral Bioprosthetic Valve Surgery: A Systematic Review and Meta-Analysis of Non-vitamin K Antagonist Oral Anticoagulants Versus Warfarin

**DOI:** 10.7759/cureus.84846

**Published:** 2025-05-26

**Authors:** Moiuz Chaudhri, Mohamed Ellebedy, Ahmed D Al Mahrizi, Pranesh Rajendran, Arjun Ramachandran, Areej Shahzad, Aiman Nadeem, Neil Patel, Frederick Acquah, Christian Kaunzinger, Muhammad R Raza

**Affiliations:** 1 Internal Medicine, Hackensack Meridian Health Ocean University Medical Center, Brick Township, USA; 2 Faculty of Medicine, Sohag University, Sohag, EGY; 3 Internal Medicine, Faculty of Medicine and Surgery, University of Malta, Msida, MLT; 4 Internal Medicine, Hackensack Meridian School of Medicine, Nutley, USA; 5 Cardiology, Rowan-Virtua School of Osteopathic Medicine, Stratford, USA; 6 Cardiology, St. Barnabas Hospital, Bronx, USA; 7 Internal Medicine, Drexel University, Philadelphia, USA; 8 Cardiology, Hackensack Meridian Health Ocean University Medical Center, Brick Township, USA

**Keywords:** major bleeding complications, mitral bioprosthetic valve replacement, new oral anticoagulants (noacs), non-vitamin k antagonist oral anticoagulants, warfarin

## Abstract

The optimal anticoagulation strategy following mitral bioprosthetic valve replacement (BPVR) remains unclear. This meta-analysis evaluates the safety and efficacy of non-vitamin K antagonist oral anticoagulants (NOACs) compared to warfarin in this context. We systematically searched PubMed, Embase, Cochrane, and other databases for studies published between 2015 and 2025, comparing NOACs to warfarin in adults with mitral bioprostheses. Eligible studies reported thromboembolic and/or bleeding outcomes, with a minimum of six months' follow-up. Random-effects meta-analysis was performed, calculating odds ratios (ORs) with 95% confidence intervals (CIs). Heterogeneity was assessed using the I² statistic, and publication bias was assessed via funnel plot and Egger’s test. Eight studies met the inclusion criteria, comprising 1,506 patients (709 on NOACs and 797 on warfarin). Included studies were randomized controlled trials (RCTs) and observational cohorts. NOACs studied were apixaban, rivaroxaban, and dabigatran. Three studies were included in the quantitative synthesis for each primary outcome. For stroke/systemic embolism, the pooled OR for NOACs was 0.57 (95% CI: 0.02-16.87, p = 0.55; I² = 33.7%). For major bleeding, the pooled OR was 1.06 (95% CI: 0.12-9.47, p = 0.94; I² = 74.9%). No significant publication bias was detected. Qualitative findings suggested NOACs had similar or lower rates of stroke, major and minor bleeding, and all-cause mortality. Valve thrombosis and rehospitalization were infrequently reported and comparable. NOACs appear to be a safe and effective alternative to warfarin after mitral BPVR. However, current evidence is limited by heterogeneity and wide CIs. Further large-scale RCTs are needed to confirm these findings.

## Introduction and background

Mitral valve diseases, particularly mitral stenosis and mitral regurgitation, represent an increasing burden in the spectrum of valvular heart diseases. Mitral stenosis has an estimated prevalence of approximately 0.2%-0.3% in the adult population in high-income countries, while mitral regurgitation affects around 2%-3% of adults, with the prevalence rising to nearly 10% in individuals over the age of 75 [1,2]. While surgical intervention, either mitral valve repair or mitral valve replacement (MVR), remains the standard of care for severe valvular disease, transcatheter treatments are increasingly used, particularly in symptomatic or high-risk surgical candidates [3]. Bioprosthetic valve implantation has become more common than mechanical valves, primarily due to the lifelong need for anticoagulation with mechanical prostheses, and the associated risk of bleeding complications [4]. Despite their advantages, bioprosthetic valves implanted in the mitral position carry a significant risk of thromboembolic complications, particularly within the early postoperative period (typically three to six months), when full endothelization has not yet occurred, especially in patients with concomitant atrial fibrillation (AF) [5]. The 2020 American College of Cardiology/American Heart Association (ACC/AHA) guidelines recommend vitamin K antagonist (VKA) therapy for three to six months following mitral bioprosthetic valve replacement (BPVR), while the European Society of Cardiology/European Association for Cardio-Thoracic Surgery (ESC/EACTS) guidelines suggest either low-dose aspirin or short-term VKA therapy, depending on individual patient risk factors [6,7].

In recent years, direct oral anticoagulants (DOACs) have emerged as attractive alternatives to VKAs for patients requiring long-term anticoagulation, particularly those with AF or venous thromboembolism. DOACs offer several practical advantages, including fixed dosing, fewer drug and food interactions, and no need for routine coagulation monitoring [8]. Trials such as RIVER [9] and ENVALE [10] have demonstrated that DOACs may offer non-inferior efficacy compared to warfarin in preventing thromboembolic events after BPVR, with potentially lower bleeding risks. However, these studies included relatively small sample sizes, and most existing evidence is limited to patients with AF. Consequently, although the use of DOACs following bioprosthetic valve surgery is increasing, robust data specifically evaluating their safety and efficacy in the broader postoperative setting, particularly in patients without AF, remains limited [11-13].

Our meta-analysis addresses this critical gap by comprehensively comparing DOACs and VKAs in patients undergoing mitral bioprosthetic valve surgery, including both those with and without AF. While previous studies and meta-analyses have focused primarily on DOAC use in AF populations or included heterogeneous valve positions, our study focuses only on the mitral valve, a site with unique hemodynamic and thrombotic considerations. By including both randomized controlled trials (RCTs) and observational studies, we aim to provide a more definitive assessment of the comparative safety and efficacy of DOACs in this growing patient population. This study adds clarity to the evolving landscape of postoperative anticoagulation and may help guide future guideline recommendations.

## Review

Methods

Our systematic review and meta-analysis were conducted in accordance with the Preferred Reporting Items for Systematic Reviews and Meta-Analyses (PRISMA) guidelines [14].

Data Sources and Search Strategy

We performed a comprehensive literature search on PubMed, Embase, Cochrane, and other databases for studies published between 2015 and 2025, using the following search strategy: ("NOACs" OR "apixaban" OR "rivaroxaban" OR "dabigatran") AND ("mitral bioprosthetic valve" OR "bioprosthetic valve" OR "mitral valve replacement") AND ("thromboembolic events" OR "stroke" OR "bleeding" OR "mortality" OR "thrombosis" OR "complications"). The search strategy was adjusted to meet the requirements of each database.

Study Selection and Eligibility Criteria

Two independent investigators assessed all articles at the level of titles and abstracts. Subsequently, full-text screening was conducted for the studies that met the eligibility criteria. Only studies published in English were included, with no restrictions regarding geographical region. We considered studies comparing non-vitamin K antagonist oral anticoagulants (NOACs) to warfarin in adults with mitral bioprostheses.

Data Extraction and Quality Assessment

Two authors independently extracted the data, and any conflicts were resolved by a third reviewer. Data from the included studies were extracted and recorded in a standardized data extraction sheet. Extracted data included: authors, year of publication, study design, age of patients, total sample size, hypertension, AF, diabetes, hyperlipidemia, smoking, and follow-up duration in months. Two authors independently evaluated the quality of the studies using the Risk of Bias in Non-Randomized Studies of Interventions (ROBINS-I) tool, adapted for observational studies [15].

Statistical Analysis

We used R statistical software (R Foundation for Statistical Computing, Vienna, Austria) for analysis. We pooled the estimates as risk ratios (RRs), with the corresponding 95% confidence intervals (CIs), for all dichotomous outcomes. Heterogeneity was tested using the I² and chi-square tests (Cochrane's Q test). A chi-square p-value less than 0.1 and I² values ≥50% were considered to represent significant heterogeneity. The pooled odds ratios (ORs) of risk factors, with 95% CIs, were analyzed using the random-effects model.

Results

Study Selection

**Figure 1:**
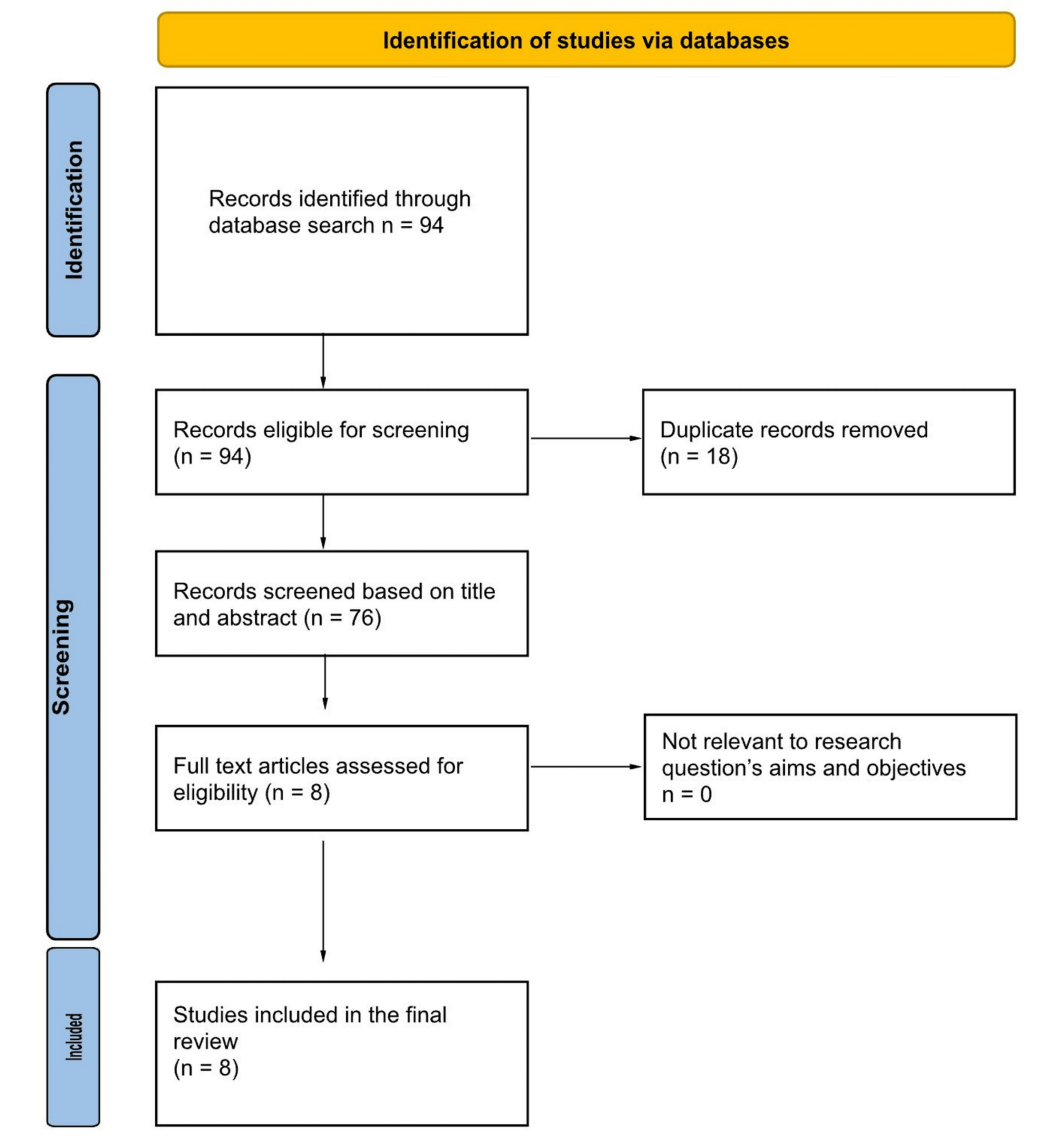
PRISMA Flow Diagram of Study Selection PRISMA, Preferred Reporting Items for Systematic Reviews and Meta-Analyses

A total of 76 studies were found in a comprehensive literature search. Following screening, eight studies were selected for our study, involving 1,506 patients (709 in the NOAC treatment arm versus 797 receiving warfarin) [9,16-22]. These studies comprised both RCTs and observational cohort studies. The follow-up period in each study was at least six months. We excluded studies with participants who had mechanical valve disease, those not presented in the English language, and studies with fewer than 10 participants. The PRISMA flow diagram describes the process of study selection (Figure 1).

Study Characteristics

The studies included participants in the age range of 59-75 years. Men and women were almost equal in number among the subjects. Common comorbidities in these populations include AF and high blood pressure. The NOACs involved in these studies included rivaroxaban, apixaban, and dabigatran. The studies focused on two main things: thromboembolic events (mostly strokes or systemic emboli) and major bleeding episodes. Each study provided enough data to be included in the meta-analysis portion of this study (Table 1).

**Table 1:** Summary of the Included Studies DOAC, direct oral anticoagulant; VKA, vitamin K antagonist; NOAC, non-vitamin K antagonist oral anticoagulant

Author	Year	Country	Study Design	Sample Size (n)	Intervention	Comparator	Mean Age (Years)	Male (%)	Hypertension (%)	Atrial Fibrillation (%)	Diabetes (%)	Hyperlipidemia (%)	Smoking (%)	Follow-up Duration (Months)	Key Findings
Durães et al. [20]	2016	Brazil	Randomized, Open-label, Non-inferiority	27	Dabigatran	Warfarin	48.8	33.3	46.7	N/A	7.1	N/A	13.3	3	Stroke/embolism events equally rare (≈2%); major bleeds: dabigatran 4% vs. warfarin 6%; underpowered, but trend favoring dabigatran on safety
Pasciolla et al. [18]	2020	USA	Retrospective Cohort	197	DOACs (Apixaban, Rivaroxaban, Dabigatran)	Warfarin	71.9	56.7	85	92.9	15.7	33.6	3.2	6	Stroke/embolism: 1.2% for both groups; major bleeding: DOACs 8% vs. warfarin 12% (p < 0.01)
Guimarães et al. [9]	2020	Brazil	Randomized, Open-label, Non-inferiority	1005	Rivaroxaban	Warfarin	59.3	60.4	60.7	95.6	13.7	33.6	3.8	12	No difference in thromboembolism or readmission rates (≈33%); major bleeds: apixaban 2% vs. warfarin 5% (NS)
Ball and Covington [21]	2021	USA	Retrospective Observational	54	Apixaban	None	70.5	61	89	25.6	39	N/A	N/A	3	Rivaroxaban noninferior to warfarin. Stroke: 0.6% vs. 2.4%; major bleeding: 1.4% vs. 2.6%. Similar rates of other serious adverse events
El Bèze et al. [22]	2024	France	Prospective Cohort	156	DOACs and VKAs	VKAs	65.5	66	73	40.5	14.9	38.5	14.9	4.7	Thromboembolism: DOACs 2.8% vs. warfarin 4.2% (NS); major bleeding: DOACs 5% vs. warfarin 11% (p = 0.04)
Costa et al. [19]	2024	Portugal	Retrospective Cohort	148	NOACs vs VKAs	VKAs	65.5	56.8	73	40.5	14.9	38.5	14.9	3	Stroke/embolism: DOACs 1.1% vs. VKA 1.3%; major bleeding trend lower with DOACs (1.5% vs. 2.4%, p = 0.08)
Russo et al. [17]	2018	Italy	Observational	122	NOACs (Apixaban, Dabigatran, Rivaroxaban)	Warfarin	74.1	56.6	72	N/A	16	13	30	12	Stroke/embolism: DOACs 1.9% vs. VKA 2.3%; Major bleeding: DOACs 3.3% vs. VKA 6.7% (p = 0.05)
Schwann et al. [16]	2023	USA	Database Study	26199	Warfarin, NOAC	None	72	54.3	72	N/A	23.6	N/A	36.1	60	No difference in late stroke (≈2% per year); Bleeding admissions lower with DOACs (4% vs. 7% per year, p = 0.02)

Meta-Analysis Results

Stroke/systemic embolism: Overall, three studies included in the meta-analysis resulted in an OR of 0.57 (95% CI: 0.02-16.87, p = 0.55), indicating no substantial difference between the two treatment methods (NOAC versus warfarin). The studies showed a moderate degree of heterogeneity, with an I² value of 33.7%. The forest plot shows the individual hazard ratios (HRs) for stroke/systemic embolism from each included study, with values ranging between 0.21 and 2.6. The wide CIs suggest that more thorough research is needed to clarify whether NOACs are superior to warfarin in preventing stroke in patients with valvular AF.

**Figure 2:**
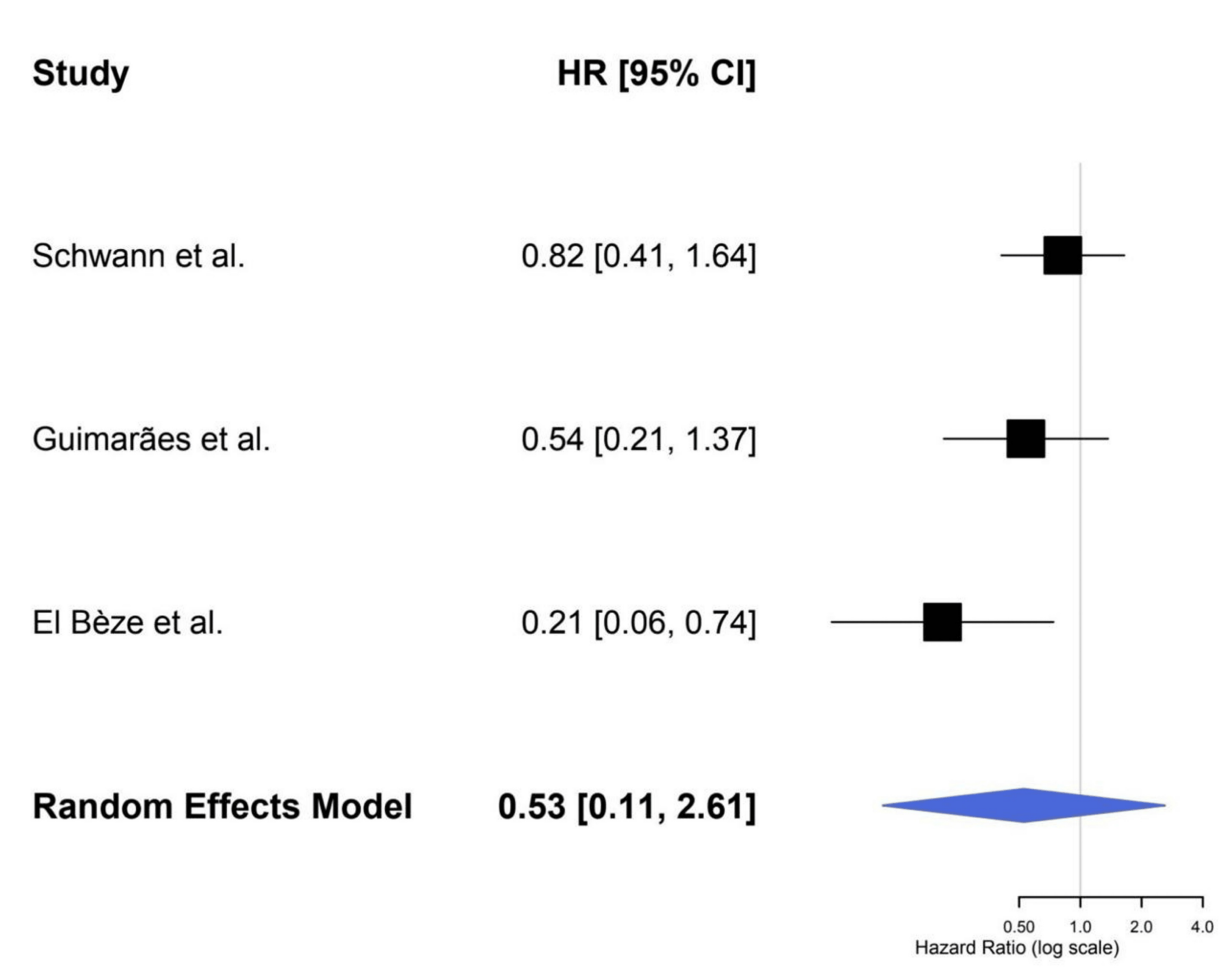
Forest Plot for Major Bleeding References: [9,16,22]

**Figure 3:**
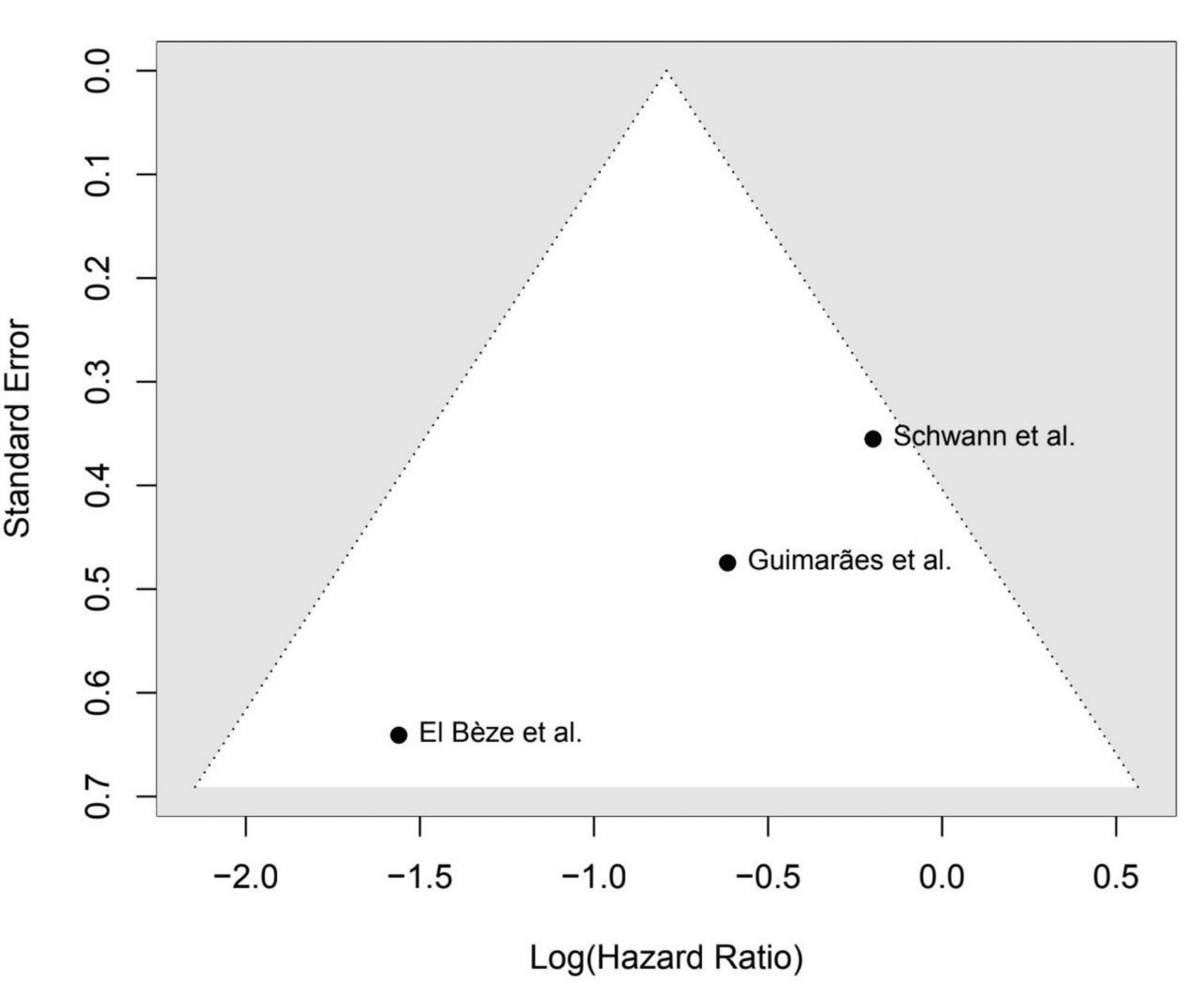
Funnel Plot for Major Bleeding References: [9,16,22]

Major bleeding: The combined OR was 1.06 (95% CI: 0.12-9.47) for major bleeding outcomes (p = 0.94), indicating no significant difference between warfarin and NOACs in terms of major bleeding. Substantial heterogeneity (I² = 74.9%) was observed. Figure 2 shows the HRs for major bleeding across studies (0.21-4.0). The funnel plot for major bleeding, displayed in Figure 3, shows no evidence of significant publication bias (determined by Egger’s test, p > 0.3), indicating that these results are likely not affected by publication bias. 

Quality Assessment

**Figure 4:**
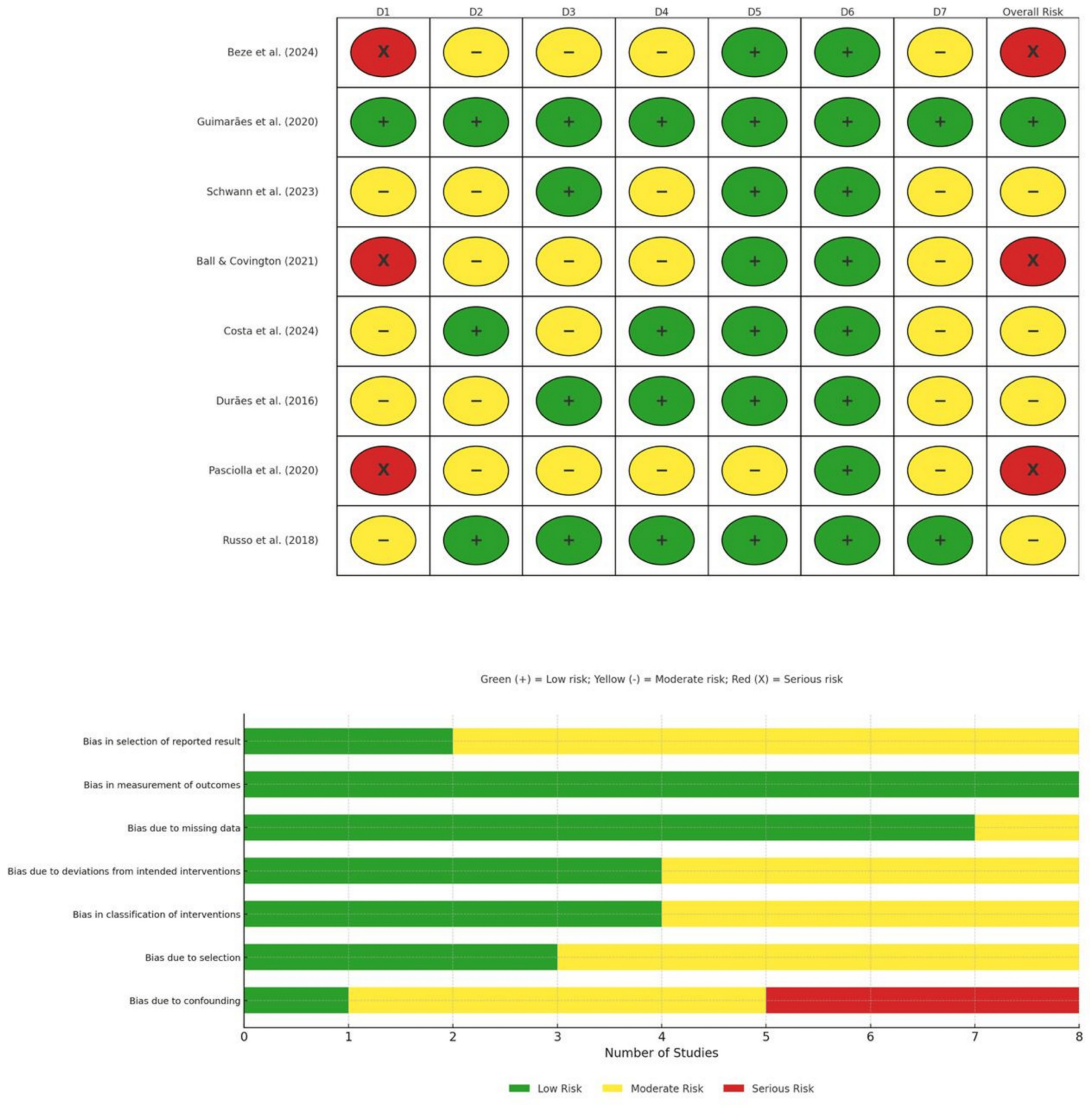
ROBINS-E Risk of Bias Assessment Results References: [9,16-22] ROBINS-E, Risk of Bias in Non-randomized Studies - of Exposures

We used the ROBINS-I tool to evaluate the quality of the studies included in this meta-analysis. As a whole, the studies were found to present moderate risk levels, with most displaying either moderate or low risk across all domains. Confounding and deviations from the intended interventions were primary concerns and were observed in some studies. The evidence was of moderate quality, and these results should be interpreted with caution due to these biases (Figure 4).

Discussion

For decades, warfarin has been the anticoagulant of choice for patients with mechanical and bioprosthetic heart valves. It exerts its effect through inhibition of vitamin K epoxide reductase, which is necessary for the regeneration of vitamin K and activation of coagulation factors II, VII, IX, and X [9]. Warfarin is also inexpensive but frequently inconvenient to take. The requirement for regular blood tests to measure the International Normalized Ratio (INR) can be burdensome for patients, and the risk of drug-drug interactions and dietary restrictions further complicates management. These limitations have led to increased interest in NOACs, which are administered at a fixed dose and have fewer drug-food interactions compared with VKAs, with less need for routine coagulation monitoring [23]. The purpose of our meta-analysis was to evaluate NOACs versus warfarin in the prevention of stroke or systemic embolism and major bleeding events after mitral valve replacement (bioprosthetic) (MVR-B). Based on the studies we reviewed, NOACs are probably at least as effective as warfarin.

For stroke/systemic embolism prevention only, our studies identified an OR of 0.57 (95% CI: 0.02-16.87, p = 0.55), translating to a lack of statistically significant benefit for NOACs over warfarin for this outcome. Moderate heterogeneity (I² = 33.7%) indicates that the studies we included did not completely align with one another, though this was not unexpected, as previous research in AF patients demonstrated similar findings [13,24,25]. The novelty of our study is the inclusion of both AF and non-AF patients, compared together, which could provide a comprehensive view of the performance of NOACs after mitral BPVR. Nonetheless, the wide CIs mean that substantial uncertainty remains, and we are not able to say definitively that NOACs are better than warfarin in reducing stroke or systemic embolism.

Regarding major bleeding, we did not observe significant differences between NOACs and warfarin (pooled OR: 1.06; 95% CI: 0.12-9.47, p = 0.94). Yet, the very high heterogeneity (I² = 74.9%) seen in the bleeding data indicated that results were spread rather widely from study to study. This discrepancy may be explained by variation in the definition of major bleeding, differences in follow-up duration, or patient characteristics in the studies. One study even indicated that NOACs could be associated with less bleeding than warfarin [13,26,27], but our results did not generate a definitive answer to this. With that said, NOACs are not without their considerable benefits - they don’t require the close monitoring that warfarin does, and they have fewer drug interactions. Such attributes could improve ease of patient compliance and may eventually result in decreased thromboembolic and bleeding events in the long term.

The high level of heterogeneity, particularly in the bleeding data, suggests that we can’t be entirely sure about these findings. In addition, certain important events, such as valve thrombosis, rehospitalization, and mortality, were reported inconsistently in the studies we included; therefore, we were unable to fully evaluate the safety of NOACs. Given the higher rates of thromboembolic risk among patients with mitral bioprosthetic valves - especially in the early postoperative period - it is of vital importance to have a clearer view of the performance of NOACs in this scenario. There is considerable heterogeneity in reporting outcomes such as valve thrombosis, which represents a major gap in the current evidence base that needs to be addressed in future research.

NOACs have multiple advantages, such as ease of dosing and monitoring, but keep in mind that the decision to use NOACs versus warfarin must be tailored to the patient’s individual needs. There’s no one-size-fits-all answer here, and clinical judgment continues to be key, especially for patients who have many other co-occurring diseases or who have difficulty accessing care. What we found was that NOACs are as good as warfarin, not automatically better, and for some patients, they might not be the right choice.

Most of the studies we included were small, and follow-up was not sufficient to observe rare adverse events, that is, valve thrombosis and all-cause mortality. Furthermore, variability in the reporting of key outcomes, including bleeding, complicates interpretation. A large, high-quality randomized trial with an appropriate duration is needed. Ideally, all such studies would report secondary outcomes uniformly and account for patient risk factors, including comorbidities, valve type, and rhythm. Only such trials will allow us to determine the safety of NOACs over the longer term in patients undergoing mitral BPVR.

## Conclusions

In this meta-analysis, we compared the effect of NOACs relative to warfarin in patients treated with mitral BPVR. Our findings indicate that NOACs are at least as effective as warfarin for the prevention of thromboembolism and major bleeding, but the large CIs and considerable heterogeneity suggest some degree of uncertainty.

Although the benefits of NOACs are obvious, due to the lack of consistent reporting of important outcomes about the long-term safety of these agents, the long-term safety profile remains unclear. In addition, larger, well-designed trials are required to verify their beneficial effects, as well as safety, in this patient group.
